# Optimizing Smoking Cessation Counseling in a University Hospital: Results and Pitfalls

**DOI:** 10.3389/frhs.2022.882964

**Published:** 2022-07-08

**Authors:** Daan L. de Frel, Veronica R. Janssen, Eline Meijer, Douwe E. Atsma

**Affiliations:** ^1^Department of Cardiology, Leiden University Medical Center, Leiden, Netherlands; ^2^National eHealth Living Lab, Leiden University Medical Center, Leiden, Netherlands; ^3^Public Health and Primary Care, Leiden University Medical Center, Leiden, Netherlands; ^4^Association Arts en Leefstijl (Physician and Lifestyle), Utrecht, Netherlands

**Keywords:** smoking cessation counseling, smoking cessation, Ask-Advise-Connect, smoking, physicians role, preventive medicine, secondary prevention

## Abstract

**Background:**

Healthcare professionals (HPs) can play a substantial role in smoking cessation counseling (SCC) but in practice often skip this task due to time constraints. This study evaluates the implementation of the rapid Ask-Advise-Connect (AAC) method in a University hospital setting.

**Methods:**

This mixed methods pre-post interventional study was performed at the Cardiology department of a University hospital and consisted of (1) a quantitative assessment of patient smoking registration and HP connection rates to external SCC from the Electronic Medical Record, (2) semi-structured interviews with 10 HPs to assess their attitudes toward AAC, and (3) a blended intervention aimed to implement AAC. The blended intervention consisted of face-to-face and online AAC psychoeducation for HPs followed-up with motivational messages on their smart pagers over a period of 6 weeks.

**Results:**

In total, 48,321 patient registrations and 67 HPs were included. Before AAC implementation, HPs assessed smoking status in 74.0% of patients and connected 9.3% of identified smokers with SCC. Post intervention, these percentages did not increase (73.2%, *p* = 0.20; and 10.9%, *p* = 0.18, respectively). Nonetheless, the vast majority (90%) of HPs feel it is important to discuss patient smoking, and view it as their duty to do so. Main barriers to AAC reported by HPs were forgetfulness and time pressure.

**Conclusion:**

This study shows that this AAC intervention does not increase Asking after smoking status or Connection of patients to SCC in a University Hospital. However, HPs hold positive attitudes toward AAC. A better understanding of the mechanisms required for optimizing HPs practice behavior is needed.

## Introduction

Worldwide, tobacco kills over eight million people yearly (1). Even though 80% of smokers is from low to middle income countries, 20.2% of Dutch adults smoked cigarettes in 2020 (2). The Dutch national institute of public health estimates that, yearly, 19.000 people in the Netherlands die due to the direct consequences of tobacco (3). This excess mortality is mainly caused by lung diseases, heart conditions and cancers.

Smoking cessation reduces the relative risk of morbidity and mortality (4, 5). Of the 10 years of life expectancy lost as a smoker, one regains 10, 9, and 6 years by stopping at 30, 40, or 50 years of age, respectively (4). Unfortunately, smoking cessation is not easy. Fewer than one in ten quit attempts from Dutch smokers is successful (6). However, studies show that a brief advice to quit smoking on medical grounds can increase long-term smoking abstinence by 47% (7). Because of this, the World Health Organization (WHO) calls on physicians and politicians to help fight the tobacco epidemic (8).

The WHO encourages physicians to assess smoking status in each patient, warn people about the dangers of tobacco and offer smoking cessation counseling (SCC) to help people to quit smoking (8). An easy and effective method of assessing smoking status, warning and offering SCC is the Ask-Advice-Connect (AAC) method. Using AAC, the physician asks about smoking, advises to quit and offers to connect the patient to an external smoking cessation program. The AAC method has a higher treatment enrolment rate than Ask-Advice-Refer (AAR) that requires patients to call the SCC provider on their own accord (9). With AAC, the contact details of the smoker are passed to the SCC provider who then contacts the smoker within 72 h (10). Despite the availability of the efficient AAC method, the majority of medical professionals still fail to connect patients to quit-smoking interventions (11). A questionnaire-based study among medical professionals of numerous departments in an academic hospital in India showed that 98% of professionals thought it was part of their role to help patients quit smoking (12). Furthermore, 95% of professionals indicated that they assessed smoking status, 94% expressed that they advised patients to quit when necessary but only 50% assisted and 28% arranged follow-up. A similar study found that cardiologists reported to Ask 73% of patients, Advise 62% of identified smokers and Refer 50% of identified smokers (13). Yet, when the patient is asked, only around one third confirms that they have been advised to quit (14). Clearly, there is a gap between practitioner reported behavior and patient reported advise rates. Thus, in order to increase smoking cessation rates we need a more thorough implementation of AAC and an objective evaluation of smoking assessment.

Known barriers to AAC implementation are the accountability of smokers, the responsibility of HPs, the role of HPs and the perceived lack of time of HPs (15, 16). Multiple methods exist to increase the efficacy of smoking cessation practices. Recent studies show that sending care providers two text messages a week can increase knowledge about the AAC model and support implementation of smoking cessation guidelines (17, 18). Whether text messages can improve implementation of the AAC method is yet unknown.

The main aim of this study was to evaluate the effect of a blended intervention aimed at HPs of the cardiology department in the Leiden University Medical Center to increase frequency of (i) smoking status assessment and (ii) connection to an external smoking cessation program. Furthermore, this study aimed to qualitatively explore the attitudes of HPs with regard to AAC and facilitators and barriers for inquiring about smoking status.

## Methods

### Setting and Subjects

**Figure 1:**
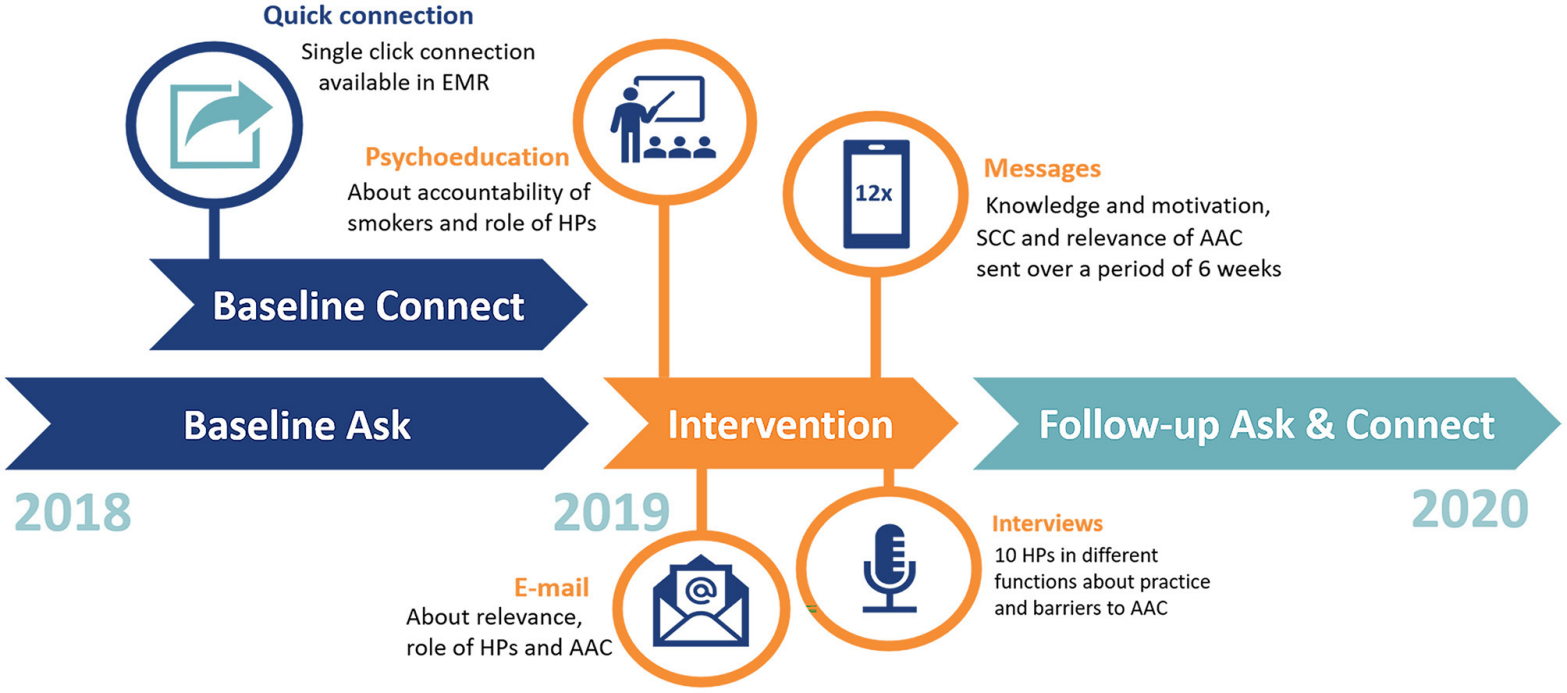
Timeline of the study. Interventions are depicted in orange. The dark blue baseline period is divided as the function to quickly connect patients to an external smoking cessation program became available in June.

This mixed methods interventional study was conducted at the cardiology department of the Leiden University Medical Center (LUMC), the Netherlands. The study period was from January 2018 until January 2020. All consultations of patients to the cardiology department were included: the outpatient clinic, the inpatient clinic, the cardiac care unit, the emergency department, the cardiac first aid, and the short stay unit. Data was collected from the electronical medical record (EMR). The year 2018 was used as baseline measurement for assessment of smoking status (Ask) and connection to an external smoking cessation program (Connect). Both were compared to the intervention period and the follow-up period (Figure 1). In total, 67 HPs (cardiologists, medical residents in cardiology, and specialist nurses) in the cardiology department of the LUMC were invited to participate in the study.

### Intervention

To increase assessment of smoking status (Ask) and connection to an external smoking cessation program (Connect), an intervention aimed at HPs was implemented from January 2019 to April 2019. The intervention was designed to familiarize HPs to the AAC method and take away potential barriers for practicing AAC. These potential barriers or determinants of behavior included: the accountability of smokers, the perceived responsibility of HPs, the actual role of HPs and the perceived lack of time of HPs. First, an email was sent to all HPs of the cardiology department that described the study and explained the role HPs could take in active smoking cessation management using the AAC method (Appendix 1). Second, a live presentation was given to the staff, updating them on the importance of smoking cessation, the accountability of smokers, the responsibility and role of HPs and the quick connecting procedure using AAC. Third, text messages on smoking facts were sent to the hospital pagers of all HPs that had provided informed consent. The aim of the text messages was to increase knowledge about the AAC method and to convey the relevance of asking after smoking status. After the example of Odukoya et al. a total of 12 messages was sent to each HP with a frequency of two text messages per week during work days (Appendix 2) (17). The messages used by Odukoya et al. were based on the precaution adoption process model. We adapted the text messages to our situation and aimed to convey importance of smoking cessation (e.g., message 3), simplicity of AAC (e.g., message 6), information about Connecting (e.g., message 7) and the benefit for the patient (e.g., message 2). The phone/pager numbers were deleted after the last text was sent.

### Outcome Measures

The quantification of the AAC implementation process in clinical practice is a challenge. After contemplating video surveillance and patient and practitioner reported data the most objective and least taxing method was considered to be data extraction from the EMR. In the EMR, HPs can score smoking status as “current,” “previously,” or “never.” If no smoking status is selected the status is “unknown.” In June 2018, a new function in the EMR became available to quickly connect (one-click) patients to an extramural SCC organization (SineFuma) that provides evidence-based, individual or group-based smoking cessation programs, which are reimbursed by health insurers. After connecting, SineFuma contacts patients within 72 h to schedule a smoking cessation program. To measure the first A (Ask) the number of patient visits in which smoking status was registered was used as an approximation of the number of patients asked about smoking status. The data concerning smoking status was divided into: “Never smoked,” “Current smoker,” “Previous smoker,” and “Unknown.” The former three were combined as “Known.” The second A (Advise) is not measured as no objective resource was available to do so. The C (Connect) was measured as the percentage of identified smokers connected to an external smoking cessation program by the HPs.

### Data Collection

All quantitative data was extracted from the EMR of the Heart Lung Center in Leiden. The variables collected are “birthdate,” “visit date,” “gender,” “place of visit,” “HP,” “smoking status,” and “connection to external smoking cessation program.” A query was used to extract the data of 53,126 patient visits. On top of the automatic query, text mining was used to identify non-parametric documentation of smoking statuses and connections. If relevant, patients that were connected to an external smoking cessation program were automatically given a “Known” and “Current smoker” status.

### Sample Size and Statistical Analyses

Sample size calculations were done using Sealed Envelope (www.sealedenvelope.com) for a superiority trial with a binary outcome and α = 5% and β = 90%. For the primary research question success was defined as “known” smoking status which was 74% in the retrospective cohort. The clinically relevant difference was set at 3% which provided a sample size of 4,316 patients per group. Chi-square tests and were used to compare the baseline group to the intervention group for both the primary and secondary outcome measure. The data were analyzed using IBM SPSS Statistics version 25. *P*-values lower than 0.05 were considered statistically significant.

### Qualitative Assessment

During the intervention period we conducted semi-structured interviews with HPs of ~30 min. The aims of the assessment were to explore attitudes of HPs regarding SCC and to quantify how many HPs experienced certain barriers. In order to identify barriers with the largest impact, participants were selected among the HPs with the lowest “Ask” and “Connect” rates in the control period. We further selected across the different professional roles (cardiologist, medical resident in cardiology and specialist nurse). This led to an interview group of ten out of 67 HPs from the cardiology department of the LUMC. The interview protocol for these semi-structured interviews is presented in Appendix 3. The interviews were summarized, discussed within the team (medical specialist, medical psychologist, and main researcher) and specific responses were quantified. This quantification aimed to visualize how many HPs shared certain attitudes (e.g., is it the duty of the HP to ask about smoking) and to pool the experienced barriers (e.g., time pressure). No qualitative coding was used.

### Ethics

The study was cleared for ethics by the Medical Ethical Committee of the Leiden University Medical Center (ref. nr. N19.027). The team adhered to the requirements for privacy and confidentiality as listed in the Privacy Statement of the Leiden University Medical Center as well as the GDPR. Written informed consent was asked of all medical HPs included in this study. Due to vast numbers of visits analyzed and relative anonymity, no explicit written consent was required from the patients included in the study. Data was pseudonymised directly after extraction and the key was handed to a trusted third party. All data was saved securely on the Research Memory of the Cardiology Department of the Leiden University Medical Center and will be saved for 15 years as legally required.

## Results

### Subjects

All 67 presently active HPs working at the cardiology department in the Leiden University Medical Center provided informed consent for this study. 52/67 (77.6%) HPs agreed to receiving motivational messages by SMS (*n* = 50) or email (*n* = 2). Reasons not to agree to receive motivational messages were “Prefer not to” (*n* = 8), “no active patient contact at the moment” (*n* = 5), “job transfer” (*n* = 2). Over a period of 6 weeks 52 HPs received 12 motivational messages. None of the participating HPs requested to terminate the messages during the study.

Data of 53,126 patient consultations were included. After removing the cardiologic consultations to other specialties (*n* = 4,804) 48,321 consultations remained. These were classified either as belonging to the baseline period conducted between January 2018 and December 2018 (*n* = 22,830), the intervention period conducted between January 2019 and April 2019 (*n* = 7,865) or the follow-up period conducted between May 2019 and January 2020 (*n* = 17,626). Some patients were included during multiple hospitable visits. As the location (e.g., outpatient clinic) or HP (e.g., physician) could be different each time, the double visits were not removed to assess the “Ask.” However, double entries were removed to assess “Connect,” so we counted the number of patients connected instead of the number of visits of patients who were connected. The patient characteristics are shown in Table 1.

**Table 1:** Characteristics of the patient visits at three timepoints.

**Characteristic**	**Baseline**	**Intervention**	**Follow-up**	**Significance**
Total number of evaluated patient visits	*22,830*	*7,865*	*17,626*	
**Age (years)**				
<30	1,247 (5.5%)	445 (5.7%)	1,024 (5.8%)	*p = 0.812*
30–45	2,034 (8.9%)	736 (9.4%)	1,595 (9.0%)	
45–60	5,543 (24.3%)	1,975 (25.1%)	4,150 (23.5%)	
60–75	9,086 (39.8%)	2,963 (37.7%)	6,999 (39.7%)	
>75	4,910 (21.5%)	1,738 (22.1%)	3,858 (21.9%)	
**Location**				
Outpatient clinic	14,558 (63.8%)*	5,218 (66.3%)	11,778 (66.8%)*	p <0.0001**
Cardiac first aid dept.	1,461 (6.4%)*	413 (5.3%)*	1,036 (5.9%)	
Coronary care unit	928 (4.1%)	294 (3.7%)	682 (3.9%)	
Inpatient clinic	2,200 (9.6%)	800 (10.2%)	1,639 (9.3%)	
Emergency room	1,540 (6.7%)	475 (6.0%)	945 (5.4%)*	
Short stay dept.	2,143 (9.4%)	665 (8.5%)	1,546 (8.8%)	
**Gender**				
Male	14,109 (61.8%)	4,829 (61.4%)	10,734 (60.9%)	*p = 0.182*
Female	8,721 (38.2%)	3,036 (38.6%)	6,892 (39.1%)	

^**^Chi-square tests were performed showing significant difference between groups on basis of location.

^*^Post-hoc analysis calculating probability scores from the adjusted residuals and using a Bonferroni correction, revealed five significant variables. The lower outpatient clinic count at baseline (p < 0.0001), the higher outpatient clinic count at follow-up (p < 0.0001), the higher cardiac first aid count at baseline (p = 0.001), the lower cardiac first aid at intervention (p = 0.002) and the lower emergency department count at follow-up (p < 0.0001) all contributed to the significant outcome of the chi-square test.

### “Ask”: Registered Smoking Status

**Figure 2:**
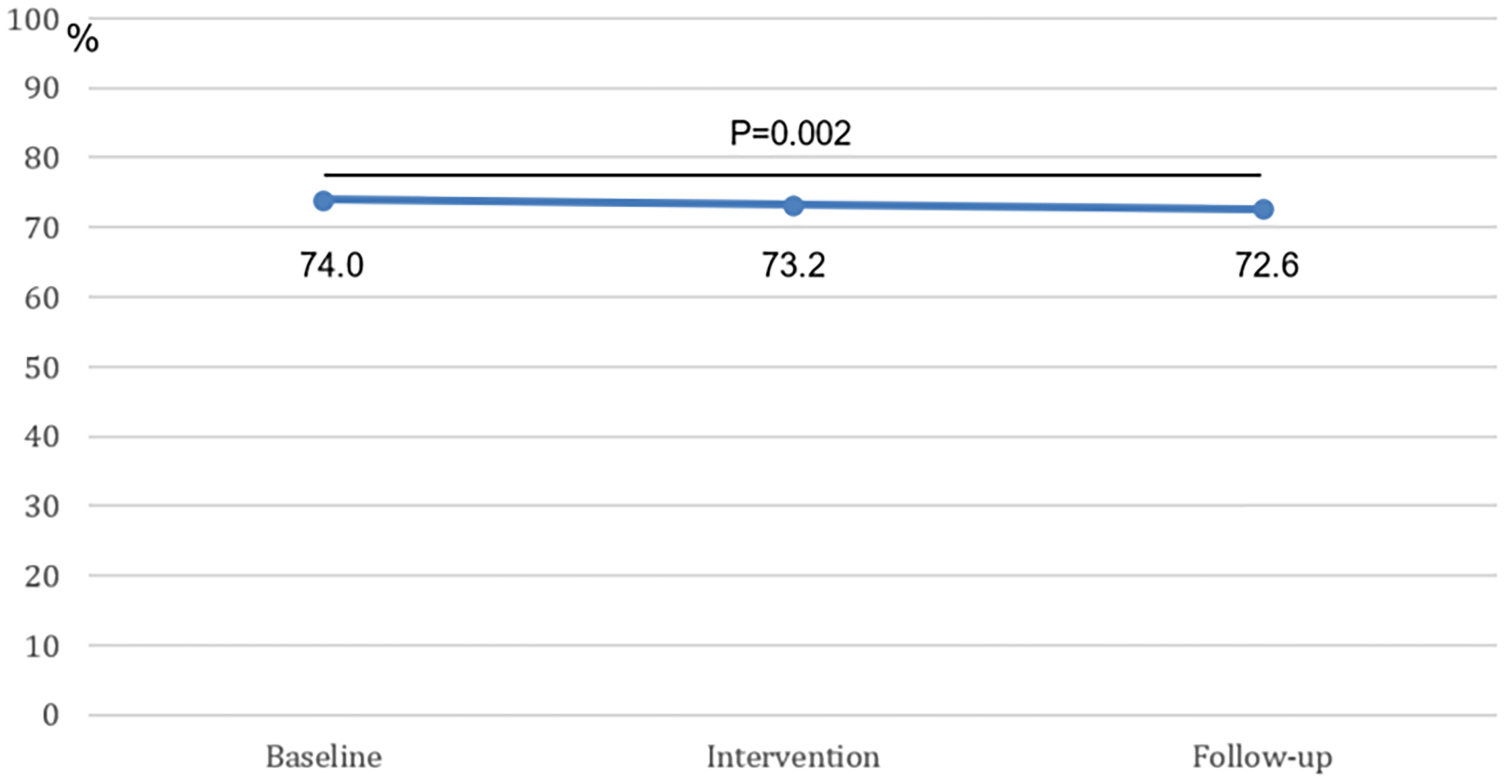
The percentage of known smoking statuses at baseline, intervention, and follow-up. The difference between the baseline period and the follow-up period was statistically significant.

During the baseline period, smoking status was known in 74.0% of patients. During the intervention no difference was observed (73.2%, *p* = 0.20). Compared with the baseline period, the percentage of visits with a known smoking status was significantly lower during the follow-up period (72.6%, (*p* = 0.002) (Figure 2). In total, out of 48,321 patient visits that were evaluated, the smoking status was “known” in 73.3% (35,441) of patient visits and “unknown” in 26.7% (12,880) of patient visits.

### “Connect”: Identified Smokers Connected to SCC

**Figure 3:**
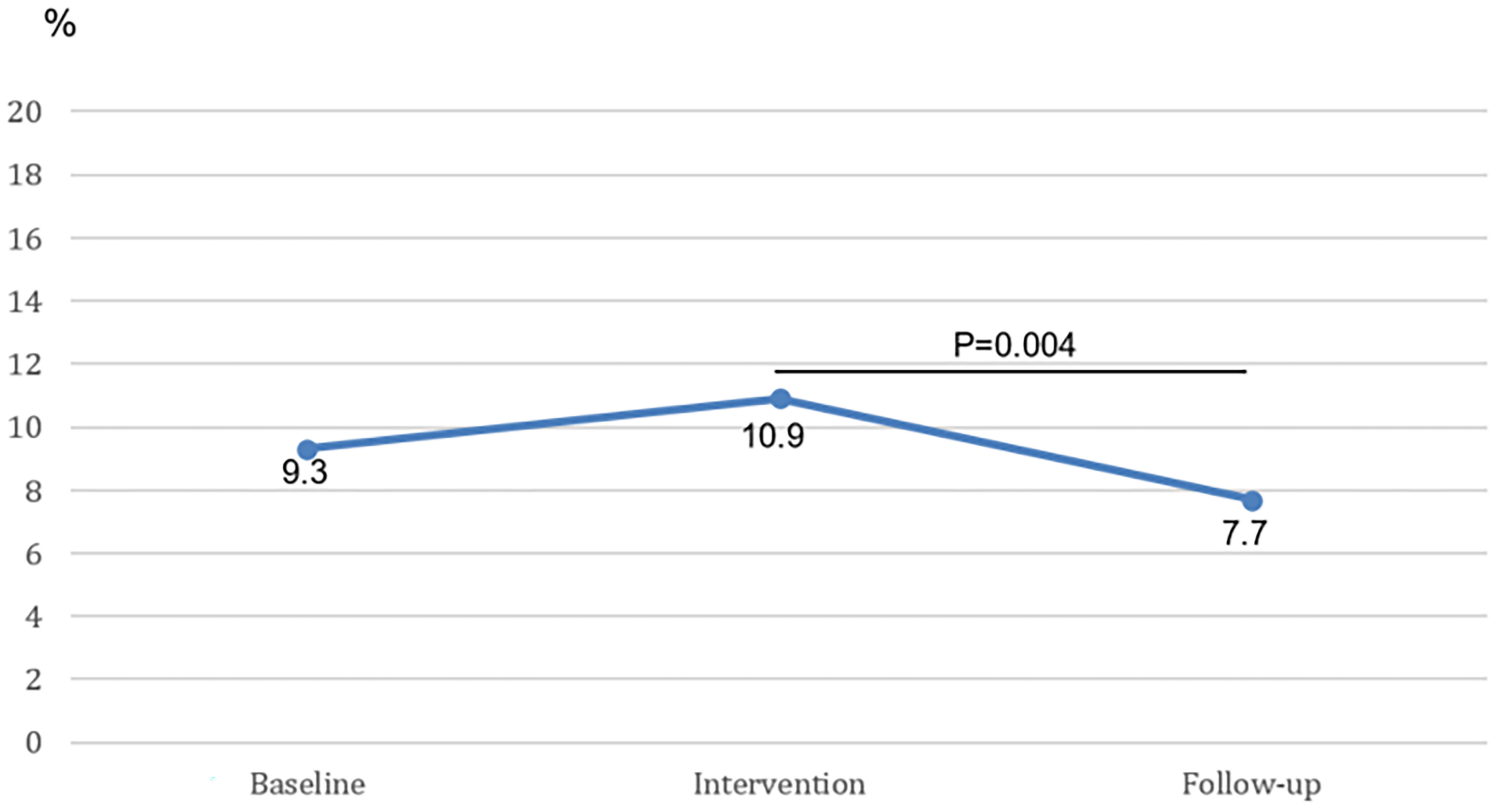
The percentage of newly identified smokers connected to external smoking cessation programs at baseline, intervention and follow-up. The difference between the intervention period and the follow-up period is statistically significant.

At baseline 156 out of 1676 (9.3%) newly identified smokers were connected to external smoking cessation programs (Figure 3). During the intervention period 97 out of 883 (10.9%) newly identified smokers were connected and during follow-up 154 out of 1996 (7.7%) smokers were connected to external smoking cessation programs. Compared with baseline, both differences were non-significant (baseline vs. intervention *p* = 0.18; baseline vs. follow-up *p* = 0.08). However, the percentage of identified smokers connected in the follow-up period was significantly lower when compared to the intervention period (10.9 vs. 7.7%, *p* = 0.004).

### Qualitative Assessment

HPs presenting the lowest Ask and Connect rates were invited to a semi-structured interview. All 10 HPs (4 cardiologists, 4 medical residents in cardiology, 2 specialist nurses) consented. Their average age was 36.4 years, 50% was female and the average years of working experience was 12.7 years.

Upon reviewing the responses of the interviews, 9/10 HPs indicated that they thought it was essential to ask every patient about their smoking status. Similarly, 9/10 HPs indicated that they perceive it as their duty to ask after smoking status and to advise cessation. For example, a cardiologist stated that “*As physician you cannot NOT discuss smoking with your patient.”* However, 6/10 HPs immediately added that smoking data should perhaps be obtained differently than by asking the patient directly as this is time-consuming during an already time-restricted patient encounter. Alternative methods suggested were via tablets in the waiting room, via questionnaires before hospital visits or via general practitioners.

Furthermore, 7/10 HPs indicated awareness of the availability of external smoking cessation programs with 5/10 reporting that they had already connected one or more patients. Barriers perceived by HPs were mainly forgetfulness (9/10), time pressure/other priorities (6/10), perceived unimportance in current consultation (4/10), or the belief that it should have been asked before by other means (electronic questionnaire or physician assistant) (2/10). One *HP stated that “In ten minutes I have to explain a diagnosis, two procedures and reach a shared decision. I do not have the extra minute for smoking status assessment.” Another mentioned that “I see patients for a second or even a third opinion, I have other things to focus on than smoking.”*

## Discussion

The purpose of this study was to evaluate the effect of a blended intervention aimed at HPs to increase the number of patients asked about smoking status, and the percentage of identified smokers connected with a smoking cessation program. The main conclusions of this study are that an implementation intervention for the AAC method in a university hospital did not increase the number of patients with a known smoking status or the number of identified smokers connected to a smoking cessation program. The results further support the hypothesis that this is most likely caused by priority issues and time pressure on the HPs involved.

This pattern of results is inconsistent with the previous literature, in which text messages have been reported to lead to a better implementation of smoking cessation strategies by HPs (17, 18). In our view, muliptle factors may explain the contrasting outcome of our study. First of all, focusing on the percentage of known smoking statuses, the lack of improvement can be partially explained by the ceiling effect. Whereas, past researchers have started at 21.2% of known smoking statuses, our study started at 74% (17). The significantly higher baseline in our study could make further improvement more challenging. A high baseline has been reported in other studies, where the percentages of patients asked about smoking were reported to be as high as 73–95% (12, 13). However, in these studies, HPs were asked how often they ask about smoking using surveys which potentially are subject to bias. This could imply that the 95% outcomes might not be attainable when the percentage of registered smoking statuses is objectively obtained from the EMR. Second, our setting is a University hospital with second opinions and complicated emergencies. It seems plausible that, with extremely rare and complex genetic diseases or in acute life-threatening situations, asking about smoking status is seen as a lesser priority. Third, with our intervention we tried to change behavior by providing knowledge about smoking cessation and the relevance of AAC to HPs. Studies show that changing people's behavior is not easily done by solely providing knowledge and relevance of a topic (19–21). For this, you also need more intensive guidance such as motivational interviewing, or environmental changes using choice architecture (22, 23). All in all, our intervention did not significantly increase the number of patients asked about smoking.

Similar explanation are relevant when focusing on the number of identified smokers connected to an external smoking cessation program. The present research showed that the implementation of a method of quick connection (one-click) accompanied by an instructive email in June 2018 led to connection rates of 9.3% of identified smokers. Which means that 90.7% of the identified smokers in the hospital is not connected to a smoking cessation program. Naturally, not every smoker is ready to quit at the time of presentation. However, taking into account that 68–80% of smokers wants to quit, there appears to be a lot of room for improvement (2, 24). On the other hand, past researchers reported similar rates (between 7.8 and 11.8%) when implementing AAC methodology in family or community clinics (9, 10). In previous literature, there are studies reporting higher rates of setting up follow-up for identified smokers (26–50%) but these were again in studies with self-reported outcome measures where HPs were asked to fill out surveys about SCC, potentially introducing self-reporting bias. In addition, these studies use the AAR method where one Refers (gives a referral note to the patient) instead of Connects (gives the patients' contact to an SCC provider) (12, 13, 25). AAR might be more easily conducted, but the percentage of connected patients enrolling into a smoking cessation program is significantly lower than with AAC (10).

The results of this qualitative assessment showed that almost all HPs who were interviewed (selected from the HPs with the lowest “ask” and “connect” rates across all professional roles) considered assessment of smoking status an essential part of their job description. These results are consistent with previous research reporting a high positive attitude toward smoking cessation strategies from HPs (26). This is an encouraging finding, as a positive HP attitude toward SCC is an essential starting point for every smoking cessation program. However, it is interesting that these were the HPs that asked about smoking the least and only half of the interviewed HPs reported having ever connected a patient to the external smoking cessation program. These findings indicate a huge gap between intention and practice of SCC. Notably, reporting bias due to social desirability may play a significant role in these interviews. More than half of HPs spontaneously mentioned that different ways should be implemented to obtain patients' smoking status and assist in smoking cessation, as their time with the patient during counseling is limited and does not allow for additional activities including SCC. Even if SCC consists of the brief AAC, HPs do not experience they have the time needed to ask about smoking. This conflicting attitude between HP's SCC ambition and implementation willingness has been reported previously, along with large differences between specialists and persisting unclarity about which HP is responsible for SCC (15).

Taken together, the results imply that the HPs agree that AAC should be integrated in hospital policies but that patients should be Asked and Connected by other means or professionals.

## Strengths and Limitations

One limitation of this study is the pre-post study design which can lead to historical bias where events unrelated to the intervention can have an influence on the outcome. Another can be the fact that only the Ask and Connect phases of AAC were objectively measured in this study. Asking after smoking status can be immediately followed by Connecting an identified smoker to a smoking cessation program, forgoing the Advice phase. Especially since HPs can find it patronizing to Advice smokers to quit, it is plausible that the Advice phase is sometimes skipped (27). We do not expect this to influence the results of this study but it should be taken into account as Advice is an integral part of AAC. As for strengths, first, the large number of patient visits included provides sufficient power to compare the percentage of known smoking statuses between the three groups. Second, all 67 presently active HPs working at the cardiology department in the Leiden University Medical Center provided informed consent for this study. This indicates that smoking cessation is considered an important aspect of care by HP's. On the same line, 52 out of 67 (75%) HPs agreed to receive messages during our study, suggesting good reach of the messages. However, no information is available about whether the messages were read and engaged with. This makes it possible that busy HPs quickly disregarded the messages. Third, the endpoints used were objective and easily obtainable leading to a low amount of missing data. Last, the qualitative assessment focused on the HPs with the lowest implementation rates, providing essential information to improve SCC effectiveness. However, it should be acknowledged that, in the future, important information could also be identified through interview with HPs with higher rates of Asking and Connecting.

In short, this mixed-methods study on 48,321 patient-consultations evaluated the effect of a blended intervention aimed at HPs. Unexpectedly, our intervention did not increase the numbers of patients asked by HPs about smoking or the number of identified smokers connected to an external smoking cessation program, most likely due to inadequate time available to HPs during patient consultations for ACC in addition to their prevailing clinical tasks. However, we found that AAC is an accepted smoking cessation strategy by HPs in a university hospital setting. Therefore, it is recommended to facilitate in-hospital AAC through an optimized logistical process involving rapid one-click connection to evidence-based smoking cessation therapy and using procedures that minimally impact the time restricted consultation with the HP without squandering the effect of a brief advice of HPs.

## Data Availability Statement

The raw data supporting the conclusions of this article will be made available by the authors, without undue reservation.

## Ethics Statement

The studies involving human participants were reviewed and approved by Medisch-Ethische Toetsingscommissie Leiden Den Haag Delft. Written informed consent for participation was not required for this study in accordance with the national legislation and the institutional requirements.

## Author Contributions

DF performed the interviews, data collection, and analyses. VJ, EM, and DA critically reviewed the manuscript in multiple stages. All authors participated in planning the study and read and approved the final manuscript.

## Conflict of Interest

The authors declare that the research was conducted in the absence of any commercial or financial relationships that could be construed as a potential conflict of interest.

## Publisher's Note

All claims expressed in this article are solely those of the authors and do not necessarily represent those of their affiliated organizations, or those of the publisher, the editors and the reviewers. Any product that may be evaluated in this article, or claim that may be made by its manufacturer, is not guaranteed or endorsed by the publisher.
