# Supplementary material for: Optimizing Smoking Cessation Counseling in a University Hospital: Results and Pitfalls
Source: Front Health Serv. 2022 Jul 8;2:882964. doi: 10.3389/frhs.2022.882964 (PMC10012747; doi:10.3389/frhs.2022.882964)
Supplement: Supplementary file 1 [file Data_Sheet_1.docx]

Appendix 1 – Mail sent to all HPs

Translated into English

Title: Study of Smoking status assessment

Best cardiologist, medical resident and specialist nurse,

This mail aims to inform you of our study about smoking within the cardiology department.

**Rationale**

Today, everybody knows that smoking is bad for health. The LUMC aims to be Smokefree as of January 1st 2020 with the help of smoking cessation counselling from SineFuma. The department of cardiology wants to lead by example on this topic. Data shows that 25% of our patients leave the hospital without being asked for smoking status. Also, of all identified smokers, 90% was not connected to external evidence-based support. This study aims to decrease these percentages by raising awareness among healthcare professionals, identifying barriers in the procedure and by overcoming these barriers.

**The study**

From January 2018 until January 2020 we will keep record of the number of patient visits with an “unknown” smoking status en the number of identified smokers connected to SineFuma. In march and April we will send messages to the healthcare professionals willing to participate. We will assess whether this type of intervention contributes to the evaluation of smoking status and the connection to SineFuma. Furthermore, a selection of healthcare professionals will be asked to participate in a short interview. This interview will focus on the barriers and facilitators in smoking cessation strategies in the hospital setting.

**What we ask of you**

1. Ask every patient about smoking and register this in the EMR. Use the AAC method: Ask about smoking, Advise to quit based on medical grounds an Connect to SineFuma. This should only take 20-30 seconds. Below this mail the procedure is explained in more detail. More information can be found on: <https://plaza.hartlongcentrum.nl/nieuws/rookvrij-ookjij/>.

If there are any questions, feel free to send an email!

1. As a reminder we would like to send messages with relevant information about smoking (cessation). This requires your consent. It will be 12 messages spread out over six weeks. Quitting is always an option. Your number and email will only be used for this study and will be deleted afterward. Please fill out the informed consent form (it will be brought to you).
2. If you have constructive feedback about the registering smoking status, connecting to SineFuma or any other remarks: Feel free to email Daan de Frel ([D.L.de_Frel@lumc.nl](mailto:D.L.de_Frel@lumc.nl)). This information will be used to optimize the process.

**Feedback**

The results of the study will be presented in May after the morning report. Tomorrow morning the study will be presented in a similar manner.

Thanks in advance for your cooperation!

Kind regards

Daan de Frel

Research student under supervision of Prof. Dr. D.E. Atsma

**Practical approach to smoking cessation advice**

**1**             **Ask about smoking**

“Do you smoke?”

**2**             **Advise to quit**

“Smoking strongly increases the risk for serious conditions of the heart, lungs and brain and increase the risk of death. I strongly advise you to quit.”

**3**             **Connect to SineFuma**

“We offer all cardiovascular patients an efficient and evidence-based smoking cessation program. The program is external and mostly covered by health insurances. If you concur I can immediately ask a professional to contact you for further information. This is a folder and you will receive extra information via the mail. SineFuma will contact you, and then you can decide if you join the program.

**Background information for Healthcare professionals**

The Heart Lung Center from the LUMC collaborates with SineFuma, an external smoking cessation program provider that works throughout the Netherlands. It provides trainings that are among the most successful quit-smoking initiatives. The goal of this collaboration is to lower the threshold for patients to join these smoking cessation programs.

**Q            What is my role?**

**A**You play a crucial role in this process because the chance of long-term absitnence increases significantly when you provide a short advice on medical grounds.

**Q            I want to connect my patient to SineFuma, how do I do that?**

**A**One can connect the patient to SineFuma via the one-click connection in the EMR via (Aanvraagmodule, tab Vragenlijsten and formulieren, check ‘Stoppen met roken’). To do this, first check the box for ‘Toestemming voor Vragenlijsten en Informatie’.

Appendix 2 – Messages sent to participants

Translated into English

| The maximum number of characters is 160 (including spaces) | | Time sent |
| --- | --- | --- |
| 1 | Welcome to the I PASS study! In the coming 6 weeks you will receive 2 messages per week with information and tips about smoking status assessment of patients. In case you do not want to receive these messages sent an email to [d.l.de_frel@lumc.nl](mailto:d.l.de_frel@lumc.nl) or respond to a text messages. Kind regards, Daan de Frel | 13-03-2019 at 11:00h |
| 2 | When a physician gives a very brief advice to stop smoking, the chance for long term abstinence increases by 47%. | *15-03-2019 at 15:00h* |
| 3 | 1 in 2 smokers dies prematurely. When one quits smoking at the age of 50, 40 or 30, one can prevent 67%, 90% and 97% of premature death respectively. | 22-03-2019 at 10:00h |
| 4 | A very brief advice to 80 people prevents one premature death. The same can be reached by providing 700 people a year with antihypertensive drugs, give 335 people angiotensin receptor blockers for one year or prescribe statins to 107 people for 5 years. | 25-03-2019 at 13:30h |
| 5 | Research in England shows that at least 12-14% of cardiac deaths are directly caused by smoking. | 28-03-2019 at 14:00h |
| 6 | With AAC; Ask, Advise and Connect, you connect a smoker to an external smoking cessation program in 2,5 minutes. “Do you smoke? I advise you to quit on medical grounds.”  “We collaborate with SineFuma, they can help you to quit. Shall I ask them to contact you? Then you can decide if you want to follow the program.” | 01-04-2019 at 16:30h |
| 7 | SineFuma offers a series of 7 group meetings after which 82% of the participants has quit. After a year, this is still 40%. Reimbursement is often once per year. | 04-04-2019 at 16:00h |
| 8 | Smoking leads to more complications and a worse outcome in surgeries. With digital help from SineFuma, these smokers can be helped quickly from their homes. | 09-04-2019 at 10:30h |
| 9 | Smoking 1 cigaret per day still gives 40-50% of the excess risk for cardiovascular disease when compared to 20 cigarettes per day. The risk for cancer does decrease by 95%. | 17-04 at 12:00h |
| 10 | Quit advice is more often given to patients who are in an advanced stage of disease, admitted to the hospital or married. Everyone benefits from quitting smoking. | 12-04 at 17:30h |
| 11 | Smokers live 9-13 years shorter. When one quits at the age of 50, 40 of 30 years, one can win back 6, 9 en 10 years respectively. | 19-04 at 10:30h |
| 12 | This is the last message, thanks for your participation. Updates from the study will be presented after the morning report. | 24-04 at 12:00h |

Appendix 3 – Interview protocol

Interview protocol (translated into English)

**Introduction**

Short summary of rationale, aims and design of study.

The LUMC aspires to be smoke free and we would like to set an example. Yet, 25% of our patients leaves the hospital without being asked about smoking status. We want to investigate how we can facilitate in stimulate asking about smoking status.

**Exploratory:**

What do you think of the idea to ask every patient about smoking?

Is there a difference between asking about smoking in the outpatient and inpatient clinic?

What do you think is the main reason not to ask about smoking?

What are other reasons?

What situations would make asking about smoking status more difficult?

**Focused questions:**

Do you have the time to ask about smoking? How lang does it take?

Do you ever forget to ask about smoking?

Do you think it worth the effort to ask about smoking?

Do you think it useful to ask about smoking?

Do you think it is your responsibility to discuss smoking?

Do you think it impacts the doctor-patient relationship?

What do you know of SineFuma (the specific external smoking cessation program provider used at LUMC)?

Extra remarks:
